# Development and Implementation of an Objective Structured Clinical Examination (OSCE) of the Subject of Surgery for Undergraduate Students in an Institution with Limited Resources

**DOI:** 10.15694/mep.2021.000097.1

**Published:** 2021-04-21

**Authors:** Omaira Rodriguez, Alexis Sánchez-Ismayel

**Affiliations:** 1 Central University of Venezuela

**Keywords:** OSCE, Assessment, International medical education, surgery

## Abstract

This article was migrated. The article was marked as recommended.

**Aim:** To develop and to test the feasibility of conducting an objective structured clinical examination (OSCE) of the subject of surgery for third-year medical students in a limited-resources institution.

**Methods:** To planning the OSCE following the Kane Validity framework. A blueprint based on curriculum was developed to design stations. A specific checklist/rubric (using google forms) was elaborated for each station. The pass/score was determined using the Modified Angoff Approach. Cronbach’s alpha was used to determine the reliability. The whole process was evaluated by assessing students’ and professors’ satisfaction using a survey.

**Results:** It was feasible to develop and implement an OSCE in an institution with limited resources. 28 students and 10 examiners participated. Both considered that the OSCE allows evaluation of the clinical competencies of the subject. They consider that this kind of assessment changed their way of studying, placing more emphasis on clinical skills. In the same way, they consider that it is, more objective, and less stressful when compared to other traditional methods. Similarly, the implementation of this strategy encourages teachers to improve teaching strategies.

**Conclusion:** It’s possible to implement un OSCE in an institution with limited resources. The incorporation of this tool has a positive impact on learning.

## Introduction

Assessment in medical education has evolved over time, nowadays, there are available a wide range of assessment tools in order to assess competences. A single tool is not enough for assessing all the components of the competencies (knowledge, abilities, skills, professionalism, attitudes). The most appropriate approach is to use a combination of assessment tools which allows us to assess adequately the students’ competencies ( Epstein, 2007; Hays, 2008; Khan, *et al.*, 2013b).

It is well known that written tests are not effective to evaluate clinical skills, traditionally, short and long case examinations have been used to evaluate students’ performance. Although these tools seem to be good methods for evaluating clinical skills, many factors result in poor reliability, such as: lack of standardization of patients between candidates, unstructured questioning by the examiners, on the other hand, in addition to the use of few clinical cases that don’t allow to assess a wide range of skills and the fact that the students are assessed by the same examiners at each case ( Ponnamperuma *et al.*, 2009; Boursicot, 2010; Khan, *et al.*, 2013b).

The Objective Structured Clinical Examination (OSCE) was introduced over 40 years ago by Harden ( Harden *et al.*, 1975), and have been used extensively for assessing clinical performance within simulated environments. The OSCE isa more valid and reliable assessment tool, based on objectivity and standardization rule which allows assessing the students across large stations of clinical cases. OSCE allows to assess communications, history taking skills, and a large range of skills and estimate students’ overall performance against standardized scoring schemes by trained teachers ( Boursicot, 2010; Boursicot *et al.*, 2011; Khan, *et al.*, 2013b).

The OSCE has become a standard for performance-based assessment in healthcare professions due to the advantages related to validity and reliability. However, the traditional assessment tools (short and long cases) are still being used widely in different parts of the world, especially in limited-resources countries, probably because of the complexity, costs, and resources necessary to implement an OSCE.

Being in a country in crisis and teach in an institution with limited resources, which oftentimes makes it difficult to move forward, however, to achieve excellence, we must adapt to improve the performance of our students.

For this reason, three years ago we decided to develop and to test the feasibility of conducting an objective structured clinical examination (OSCE) of the subject of surgery for third-year medical students in a limited-resources institution.

## Methods

Planning of the OSCE was done following the Kane Validity framework ( Kane, 2013; Cook *et al.*, 2015). We designed an OSCE for third-year medical students (Subject Surgery). It is a summative test and the goal of the test is the assessment of clinical skills, such as, history-taking, physical examination skills, image interpretation, critical thinking, and communication skills.

The OSCE stations were designed and reviewed with the teachers of the subject. We selected components of the competence to be assessed based on the curriculum. A blueprint was prepared in order to include selected competences ( Table 1, Table 2)

**Table 1:** Blueprint 1

	HISTORY TAKING	CLINICAL EXAMINATION	ASSESSMENT/DIAGNOSIS	MANAGEMENT	COMUNICATION SKILL
NECK		X			
CHEST		X			
ABDOMEN		X			
LIMBS		X			
ABDOMINAL PAIN	X	X	X	X	X
BREAST CANCER	X			X	X
THYROID NODULE			X	X	
GASTROINTESTINAL BLEDDING			X	X	
ABDOMINAL WALL			X	X	

**Table 2:** Blueprint 2

STATION	CLINICAL CONTEXT	ORGAN SYSTEM	CLINICAL PRESENTATION	UNDERLYING CONDITION	STUDENT TASK
1	Emergency department	Gastrointestinal	Abdominal pain	Cholecystitis	-History taking -Integration of knowledge -Clinical examination -Assessment/diagnosis -Communication skill
2	Office	Breast	Control	Healthy	-Integration of knowledge -Communication skill
3	Emergency department	Gastrointestinal	Abdominal pain	Appendicitis	-Integration of knowledge -Management -Clinical examination -Assessment/diagnosis -Communication skill
4	Office	Neck, chest, abdomen, limbs	Asymptomatic	Healthy	-Physical examination
5	Clinical case (Video-Image)	Neck, gastrointestinal, abdomen, abdominal wall.		Thyroid cancer Bowel obstruction Gastrointestinal bleeding Hernia	-Integration of knowledge -Management -Image interpretation

We decided to design 5 stations:

• ***Four observed stations*** with standardized patients in order to assess: clinical history taking, diagnosis, differential diagnosis, directed physical examination, work plan, interpretation of images, integration of knowledge and communication skills.
• ***One unobserved station*** with electronic clinical case to assess integration of knowledge and interpretation of images.

A specific checklist/rubric was elaborated for each station. We used google forms (Google Inc.) to do checklist which is completed online by teachers using their smartphones, in this way the collection and analysis of data, and therefore the test results analysis are facilitated. The highest possible score for each station was 100.

The method used to determine the pass score was Modified Angoff Approach ( McKinley and Norcini, 2014), for this purpose a group of panelists consisting of six teachers of the subject were selected: 4 females and 2 males, ages between 30 and 43 with teaching experience of 2-13 years. The objective of the evaluation was previously explained to them.

Later, the selected methodology to determine the pass/fail was explained and discussed, as well as the characteristics of the borderline group (minimum necessary competencies that a third-year medical student must have in the subject of surgery).

The materials and checklists of each station were sent to all the judges in digital format with a concise explanation about what they needed to do. At each station, the panelists had to define the percentage the borderline group must have to pass the station. Subsequently, all the judges reviewed the result, if there was a discrepancy greater than 15% in the stations, they could discuss and re-qualify, then the average of each station was calculated. Finally, the pass score was determined using the average of the stations. ( Table 3)

**Table 3:** Standard Setting: Modified Angoff. Passing Score

	RATER 1	RATER 2	RATER 3	RATER 4	RATER 5	RATER 6	RATER AVERAGE
**STATION 1**	55	60	55	54	52	54	55
**STATION 2**	60	60	60	55	54	52	56.8
**STATION 3**	53	55	50	52	60	50	53.3
**STATION 4**	60	55	50	55	52	60	55.3
**STATION 5**	50	54	60	54	55	50	53.8
**PASSING SCORE**							**54.8**

For the implementation of an OSCE it is necessary to have adequate space and material, and human resources. We don’t have institutional financing, for this reason we carry out this evaluation with limited and self-financed resources. ( Table 4)

**Table 4:** Resources

RESOURCES		Cost ($)
**MATERIAL RESOURCES**		
**Office and furniture**	Offices and furniture of the department were used	-
**Office material**	Sheets, cardboards, pens	20
**Laptops and iPad**	5 Teachers devices were used for technology-enhanced station.	borrowed
**Walkie talkies**	4 devices were used for the activity.	borrowed
**Cellphones**	10 devices were used	teacher’s cell phones
**HUMAN RESOURCES**		
**Coordinator**	Professor	-
**Administrative team**	Professor, volunteers’ students and secretary.	-
**Logistic team**	Professor, students	-
**Training Teachers**	Professors	-
**Standardized patients**	Volunteer students	-
**SNACK AND DRINKS**	Snack and drink for staff and SP	60

### Standardized patients

We developed scripts for standardized patients, detailing all the aspects for the role they will play. We don’t have actors so we trained volunteer medical students and residents as standardized patients.

The script have all details about: personalinformation such as name, age, sex, and occupation. Illness related history **,** including pertinent positives and negatives, medical and social history, they were also trained to answer unanticipated question with “no” or I “don’t remember”, and to simulate reaction to stimuli like signs of peritoneal irritation.

### Training teachers

We perform a meeting with the instructors in order to explain them the objective and the whole process of the OSCE.

Instructions werewritten for each station for both teachers and students explaining the objectives of each station and the time required for each of them.

A pilot test was carried out to allow students and teachers become familiar with the dynamics and rubrics. It allowed us to determine the appropriate time for each station.

The stations were placed in separate nearby environments to facilitate rotation of the students.

Time for each station was: station 1 (10 min), station 2 (15 min), station 3 (15 min), station 4 (5 min), station 5 (10 min).

The reliability of the exam was determined by calculating the Cronbach’s alpha for each of the scoring tools. The data were analyzed using IBM SPSS Statistics for Macintosh, Version 25.0 (IBM Corp; Armonk, NY).

## Results

It was feasible to develop and implement an OSCE in an institution with limited resources. Twenty eight students and ten examiners participated in it.

On the day of the exam, the instructions were briefly reviewed with the instructors, students and SP separately. Written instructions were given to students and teachers.

We used duplicate stations in order to reduce the test time, fatigue of the SP and teachers. Each station was identified with a color and a number. A card was given to the students to check the stations through which they had already rotated. The total time of the test was 2:30 hours.

Finally, the debriefing was carried out at the end of the test where all the teachers met with the students to clarify doubts, share experiences as well as suggestions and ideas to improve. Subsequently, a survey was conducted in digital format to assess the quality of the exam, impact on learning and degree of satisfaction.

The whole process was evaluated through assessing students’ and professors’ satisfaction using a survey. ( Table 5)

**Table 5:** Perception of examiners and examinees regarding congruence, feasibility, acceptability and educational impact of OSCE

	Percentage of strongly agree and agree ( N:10)	Percentage of strongly agree and agree (N:28)
	Examiners (%)	Examinees (%)
**Congruency**		
The task can assess the competencies	100	92,8
**Feasibility**		
Adequate briefing before examination	100	89,2
Time allocated for completing the stations was sufficient	90	89,2
Instructions for examinee is clear	100	100
Well Organized OSCE	100	100
**Acceptability**		
OSCE is appropriate to assess the clinical competence	100	92,8
**Educational Impact**		
The application the OSCE Change the way examinees study	100	89,2
OSCE stimulates examines to learn clinical competencies	100	100
OSCE is less stressful than other kind of test	90	85,7
The OSCE evaluates the abilities and skill acquired.	100	100
More objective assessment	100	92,8
OSCE implementation encourages better teaching	90	85,7

In general, both teachers and students considered that the OSCE is an assessment tool that allows evaluation of the clinical competencies of the subject. It is important to highlight that the incorporation of this tool has had a positive impact on learning. Both students and teachers consider that this kind of assessment changed their way of studying, placing more emphasis on necessary clinical skills for the acquisition of competences. In the same way, they consider that this kind of assessment is more objective, and less stressful when compared to other traditional methods. Similarly, the implementation of this strategy encourages teachers to improve teaching strategies.

Cronbach’s alpha was used to determine the reliability of the OSCE. The internal consistency was good with a Cronbach’s α >0.7 (range, 0.71-0.83), which indicates correlations among the items in the scale. ( Table 6)

**Table 6:** Cronbach’s alpha

	Cronbach’s alpha
**STATION 1**	0.717
**STATION 2**	0.720
**STATION 3**	0.830
**STATION 4**	0.834
**STATION 5**	0.719

This was our initial experience organizing this kind of assessment. It has been a radical change in the way we perform assessment of the subject. At the beginning, it was not easy to convince teachers to adopt this tool due to had never been used in the institution. After reviewing the literature and multiple meetings, we managed to create a working group to design and plan the OSCE. From this moment on, we have used this assessment tool, which has been widely accepted by both students and teachers.

## Discussion

The OSCE is a valid and reliable assessment tool which allows assessing the performance’s students within environment control. To guarantee the quality of the OSCE we must guarantee that it has high levels of validity and a high degree of reliability ( Pell *et al.*, 2010). Validity allows us to ensure that evaluation measures what it is intended to measure. In this way, we must know how to guarantee the validity of the assessment. It is well known that validation has evolved from different types of validation to a unitary concept in which different sources of validity are explored such as the one proposed by Messick (cited in Cook *et al.*, 2015) which consists of 5 different sources of validity evidence, and more recently the Kane’s framework that focuses on four steps: scoring (translating an observation into one or more scores) generalization (using the score as a reflection of performance in a test setting) extrapolation (using the score as a reflection of real-world performance) and implications (applying the score to inform a decision or action) ( Kane, 2013; Cook *et al.*, 2015).

The first thing to do when developing an OSCE is to determine the purpose of the evaluation in our case it is a summative assessment which provides evidence for the implications stage of kane’s model ( Kane, 2013). It is important to note that the OSCE cannot be used to evaluate the entire content of the subject, so it must be selected which are the components of the competencies that we want to evaluate ( Daniels and Pugh, 2018). To ensure that the evaluation meets the stated objective, the ideal is to perform a blueprinting ( Coderre, Woloschuk and McLaughlin, 2009). This helps us to have a proper sample of the domains (generalization). Usually, the time of the stations varies from 5-10 min depending on the task to be evaluated ( Khan, *et al.*, 2013b).

Regarding the number of stations, it is recommended an adequate number to evaluate the construct of interest. Testing students through a greater number of stations increases reliability and an appropriate test length time ensures that candidates’ overall performance is reliably assessed ( Newble, 2004; Khan, *et al.*, 2013b). In lower stakes assessment developed locally could be 8-10 stations while high stakes assessment may require more stations to achieve acceptable reliability ( Khan, *et al.*, 2013b). In our test we only use 5 stations, because it is an OSCE for a single subject (surgery), we consider that they are enough to assess the components of the selected competencies, however, to increase the reliability of the test we could increase the number of stations.

According to Daniel and Pugh (2018) it is very important to develop properly the cases, to ensure that they represent authentic clinical problems. The cases were developed by a group of experts who determined that they adequately represent clinical problems, with an adequate difficulty for the level of the students. Similarly, we observed that the time to complete the task was sufficient. (Kane’s stage extrapolation). Instructions must be given to students including pertinent information about the case, the task to be carried out as well as the time to complete it.

For the assessment to be objective, it is important to use rubrics or checklists that allow us to evaluate observable behavior ( Daniels and Pugh, 2018), in our case we created and used specific checklists for each station. The instructors received a pre-test training where information about OSCE objective, the learning level, and use of the checklists was provided. Later they became familiar with them during the pilot.

The use of standardized patients allows students to demonstrate their clinical skills by reducing variations between SPs. In this way, the evaluation is fairer since the students are evaluated by the same cases. Therefore, it is important to write a script for the SP that specifies all the medical history data related to the disease, including the date of onset of symptoms, relevant negative or positives, etc. Likewise, they should receive training to simulate physical examination findings ( Furman, Smee and Wilson, 2010; Daniels and Pugh, 2018).

The integrity of the data collection must be ensured. This provides evidence that the test reflects what has been observed (Kane’s scoring stage) ( Daniels and Pugh, 2018). We created checklist / rubrics online with google form in order to facilitate the data collection. Many centers use eOSCE systems, these facilitate data storage and analysis as well as reduce errors and can increment the quantity and quality of feedback ( Meskell *et al.*, 2015; Denison, Bate and Thompson, 2016).

Establishing the pass/fail score is essential to determine if the student is competent or not. There is no ideal standard setting method, the most frequently used for OSCE are the criterion-referenced method such as Angoff, Borderline group, and borderline regression ( McKinley and Norcini, 2014). We use Angoff’s method, as it is an easy method to understand and implement. However, many authors recommend the borderline regression method because it is a straightforward method, based on actual performance of all examinees, it uses the judgments of expert examiners. Another important advantage is that it can be used to generate metrics to evaluate the quality of an OSCE such as the *R*2 coefficient, the adjusted value of *R*2, and the inter-grade discriminatione. All these data will allow us to perform a more optimal measurement of assessment ( Wood, Humphrey-Murto and Norman, 2006; Pell *et al.*, 2010; Mortaz Hejri *et al.*, 2013).

One important point is to decide is whether the passing score will be compensatory or conjunctive. It is done based on overall score of the stations (compensatory) or if you need to pass a certain number of stations (conjunctive) ( McKinley and Norcini, 2014). In Our OSCE the standard would be considered compensatory.

Another important source of validity evidence is related to the generalizability of results, so it is necessary to analyze the psychometric properties of the OSCE. Score reliability is an important source of Validity. Cronbach’s alpha is frequently used to measure overall station reliability and to determine which stations were not properly designed. Because in OSCE there are many related factors that can be source of error (students, item, raters, station, etc.) the G-Theory is preferred by many authors to calculate reliability as well as to determine various sources of error ( Kane, 2013; Cook *et al.*, 2015; Daniels and Pugh, 2018).

According to Godfrey Pell *et al*. (2010), the quality of the OSCE should be measured by analyzing a set of metrics (Cronbach’s alpha, coefficient of determination R2, intergrade discrimination, number of failures, variation between group, etc) since we can obtain a more realistic picture of quality of assessment, we must identify which are the strengths and weaknesses of the assessment tools.

It is well known that assessment is a fundamental part of learning. It is important to determine the impact of the OSCE evaluation on the learning. Often times the student focuses on learning to pass the exam and not in learning objectives. For this reason, it is important to emphasize the importance of acquiring skills for performance in medical practice. Likewise, ensure that the learning objectives are aligned with the content of the evaluated clinical skills ( Boursicot, 2010; Khan, *et al.*, 2013a).

The feedback provided by students and teachers is an important step that can be used to improve the quality of the stations and the organization for future exams. knowing the opinion of the students concerning the evaluation process is essential since it will allow us to determine failures such as lack of clarity in the instructions, a difficult task, insufficient time to carry out the task assigned in the stations, etc. Similarly, the opinion of the teachers provides us with valuable information about the organization and eventual issues that may arise during the assessment ( Khan, *et al.*, 2013a).

In the same way, provide students with adequate feedback is a fundamental part of any kind of assessment. The students should know their strengths and weaknesses, to progress in the learning process. The feedback helps to promote the acquisition of skills and drives professional growth and development ( Van De Ridder *et al.*, 2008; Khan, *et al.*, 2013a). For this reason, teachers need to be trained to ensure that feedback has a positive impact on learning.

Organizing and planning an OSCE is not an easy task, it consumes more time and effort, it involves a more complex logistics than another kind of assessment. Ideally, when we incorporate a new evaluation method in our programs, we should count on the support of an experienced evaluation team to guide the process. Once the OSCE has been successfully implemented, continuous evaluation is necessary to ensure the quality of the process ( Khan, *et al.*, 2013a; Daniels and Pugh, 2018).

## Conclusion

Although being in an institution with limited resources indeed makes it difficult to carry out complex assessments that involve an organizational structure, adequate infrastructure, and enough human and material resources, it is also true that we can and we must adapt. The incorporation of this tool has a positive impact on learning.

This first step is a small sample of what we can achieve to improve the quality of assessment and student performance.

## Take Home Messages

• Planning of the OSCE following the Kane Validity framework helps to guarantee that evaluation measures what it is intended to measure.
• The OSCE is a complex assessment that involves an organizational structure, adequate infrastructure, and enough human and material resources, but it’s possible to implement an institution with limited resources.
• The OSCE is an objective assessment that must be incorporated to assess clinical competence in all medical schools.
• It is an assessment tool that stimulate students to learn clinical competence and encourages better teacher.
• The incorporation of this tool has had a positive impact on the learning process.

## Notes On Contributors

**Omaira Rodriguez**, MD. Aggregate Professor. Department of Surgery III. “Luis Razetti” medical school. Central University of Venezuela. Caracas, Venezuela. ORCiD: https://orcid.org/0000-0002-0322-8073

**Alexis Sánchez-Ismayel**, MD, MSc.Associate Professor.Department of Surgery III. “Luis Razetti” medical school. Central University of Venezuela. Caracas, Venezuela.
