# Supplementary material for: Development and Implementation of an Objective Structured Clinical Examination (OSCE) of the Subject of Surgery for Undergraduate Students in an Institution with Limited Resources
Source: MedEdPublish (2016). 2021 Apr 21;10:97. [Version 1] doi: 10.15694/mep.2021.000097.1 (PMC10939656; doi:10.15694/mep.2021.000097.1)
Supplement: Peer review history

# Reviewer report: Reviewer response for version 1

**DOI:** 10.21956/mep.18902.r27008

**Published:** 2021-07-13

**Authors:** Richard Hays

**Affiliations:** 1 James Cook University

This review has been migrated. The reviewer awarded 3 stars out of 5 In accepting the authors' statement that this work was done in a relatively resource poor context, this article describes well the process of selecting OSCE station content. The OSCE was a new method for this program and both examiners and candidates evaluated the event highly. Other programs considering introducing OSCEs could benefit from reading this, but there are some additional considerations to work through. Thinking of the 'assessment utility' concept (Van der Vleuten), the sampling framework should achieve validity. I do not believe that 5 stations reflects validity as there are so many clinical skills that could be measured. More is better in this situation and most OSCEs have 12-16 or more stations, allowing roader sampling. Reliability is also likely to be low with only 5 judgements - this is not the primary purpose of the article but is important. Increasing station numbers creates problems for logistics but educational impact is improved where candidates see clear links between assessment tasks and interacting with patients (simulated and real). I agree that this about competence and that performance measures are becoming increasingly important, but taking this careful and deliberate approach to sampling is sound when developing an OSCE.

Reviewer Expertise:

NA

No decision status is available

# Reviewer report: Reviewer response for version 1

**DOI:** 10.21956/mep.18902.r27007

**Published:** 2021-07-12

**Authors:** Balakrishnan Nair

**Affiliations:** 1 Centre for Medical Professional Development and University of Newcastle

This review has been migrated. The reviewer awarded 4 stars out of 5 OSCE is for competency assessment and not for performance assessment . It can assess clinical skills in a structured way , which is its strength. It can not measure professionalism , team work etc in a proper way. These should have been discussed in the introduction. Methods are clear and conclusions are right. I think doing mini- clinical evaluation and case based discussions may be cheaper ways of assessing students . Overall it is a well written paper

Reviewer Expertise:

NA

No decision status is available

# Reviewer report: Reviewer response for version 1

**DOI:** 10.21956/mep.18902.r27006

**Published:** 2021-04-24

**Authors:** Rajaei Sharma

**Affiliations:** 1 College of Medicine and Health, University of Exeter

This review has been migrated. The reviewer awarded 2 stars out of 5 This is an important topic for general medical education, one that may be overlooked given the assumed benefit. It is likely specifically relevant here given the global readership of MedEdPublish. The introduction offers little background as to the relevance of OSCE’s in assessment. It would be useful to have a more depth to the introductory discussion; why have they become the standard for assessment, what are the alternatives, has there been any other recent work on this and why is this study specifically relevant now.The methods section is well written and offers great detail. This would be easily replicated in another setting. It is good to see that statistical evaluation was used as appropriate through-out the implementation. Given so many assessors were used relative to the number of students, it would be important to explore how marking was standardised. The results are clear, but it may have been useful to also include free-text or quotes from all participants involved.The discussion is also well written and makes good reference to the relevant literature. Given the comparative length of the discussion, a longer conclusion would be useful for the reader to summise the key points, we should not rely on the take home messages. It may also be worthwhile for the authors to suggest next steps in research or identify voids in the current literature.Overall, this is a good piece of research and may serve to be useful for readers in similar situations seeking to improve the assessment capabilities of their medical school. Further revision would likely make this a very nice piece to read and give a slightly more holistic view.

Reviewer Expertise:

NA

No decision status is available
